# In-hospital outcomes after acute myocardial infarction with obstructive coronary artery disease in critically ill patients hospitalized for non-cardiac disease

**DOI:** 10.1186/s13613-023-01188-9

**Published:** 2023-09-19

**Authors:** Morgan Roué, Alexis F. Guédon, Nathanaël Lapidus, Keyvan Razazi, Geoffroy Hariri, Elise Morawiec, Cyrielle Desnos, Stéphane Ederhy, Ariel Cohen, Armand Mekontso Dessap, Muriel Fartoukh, Vincent Labbé

**Affiliations:** 1 Service de Médecine Intensive Réanimation, Département Médico-Universitaire APPROCHES, Hôpital Tenon, Assistance Publique-Hôpitaux de Paris (AP-HP), Sorbonne Université, Paris, France; 2 Sorbonne Université, Public Health Department, Saint Antoine Hospital, AP-HP, Paris, France; 3 Service de Médecine Intensive Réanimation, Hôpitaux Universitaires Henri Mondor-Albert Chenevier, Département Médico-Universitaire Médecine, AP-HP, Créteil, France; 4 Université Paris Est, Groupe de Recherche Clinique GR05 CARMAS, Institut Mondor de Recherche Biomédicale, INSERM, Créteil, France; 5 Service de Médecine Intensive Réanimation, Hôpital Saint-Antoine, AP-HP, Sorbonne Université, Paris, France; 6 Service de Médecine Intensive Réanimation, Hôpital La Pitié-Salpêtrière, AP-HP, Sorbonne Université, Paris, France; 7 Department of Cardiology, UNICO Cardio-Oncology Program, Hôpital Saint-Antoine, AP-HP, Paris, France; 8 INSERM U 856, Paris, France; 9 Sorbonne Université, UMR-S ICAN 1166, Paris, France; 10 Service des Soins Intensifs, Hôpital Universitaire Bruxelles, Université Libre de Bruxelles, Brussels, Belgium; 11 Sorbonne Université, INSERM, Institut Pierre Louis d’Epidémiologie et de Santé Publique IPLESP, AP-HP, Paris, France

**Keywords:** Acute myocardial infarction, Intensive care unit, Ischemic risk, Bleeding risk, Outcomes, Coronary artery disease

## Abstract

**Background:**

Acute myocardial infarction (AMI) is one of the major cardiac complications in patients hospitalized in the intensive care unit (ICU) for non-cardiac disease. A better knowledge of ischemic and bleeding risks in these patients is needed to identify those most likely to benefit from specific cardiac management. We therefore assessed the incidence and predictors of a composite outcome of severe ischemic event (AMI recurrence, ischemic stroke), major bleeding, or all-cause death in this setting.

**Methods:**

In this multicenter retrospective study, all consecutive adult patients admitted for non-cardiac disease to four French university hospital ICUs between January 2012 and December 2018 who had an AMI with obstructive coronary artery disease (OCAD) during the ICU stay were considered for inclusion. AMI with OCAD was defined as an elevated cardiac troponin value associated with at least one sign (clinical, electrocardiographic, or echocardiographic) suggestive of myocardial ischemia and presence of OCAD on coronary angiography. The primary endpoint was in-hospital occurrence of the composite outcome.

**Results:**

Ninety-six patients [median age 69 years, 22 women (23%), 59 with sepsis (61%), 35 with ST elevation (37%), median sequential organ failure assessment (SOFA) of 8 on the day of AMI] were included. The median peak cardiac troponin value was 131 (IQR 44–303) times the upper reference limit. Dual antiplatelet, therapeutic anticoagulation, and early mechanical reperfusion therapies were administered in 61 (64%), 68 (71%), and 47 (49%) patients, respectively. The composite outcome occurred in 48 (50%) patients. Severe ischemic events occurred in 17 (18%) patients and major bleeding in 26 (27%) patients; 26 patients (27%) died in the hospital. AMI management was not significantly different in patients with and without the composite outcome. A history of arterial hypertension (HR 2.05, 95% CI 1.01–4.16) and high SOFA score at the time of AMI (HR 1.07, 95% CI 1.00–1.15) were independent risk factors for the composite outcome.

**Conclusions:**

Patients who have an AMI with OCAD during an ICU stay for non-cardiac disease are at risk of a composite outcome of severe ischemia, major bleeding, and death. A history of arterial hypertension and high SOFA scores were independent hazards for poor prognosis.

**Graphical Abstract:**

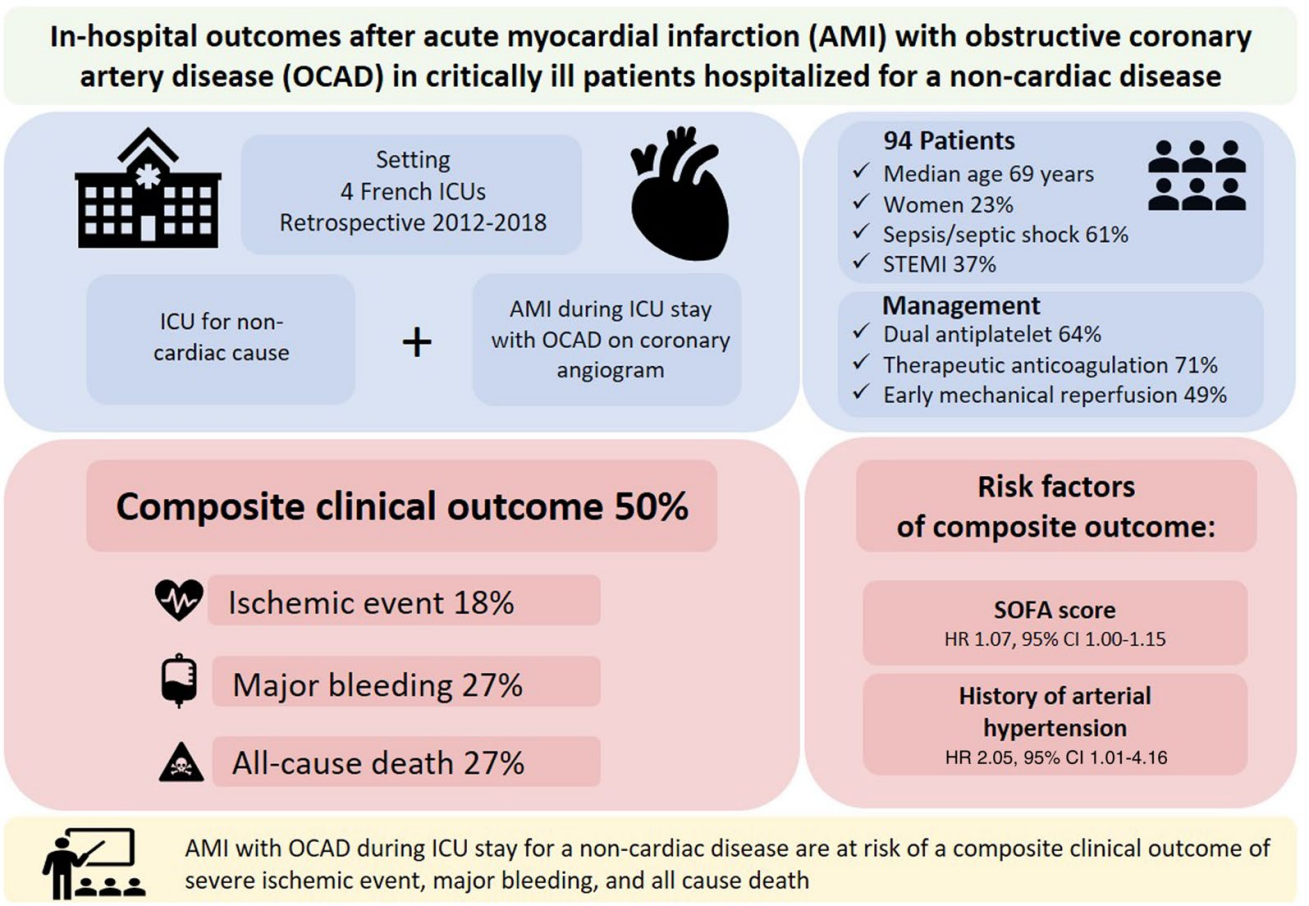

**Supplementary Information:**

The online version contains supplementary material available at 10.1186/s13613-023-01188-9.

## Background

Acute myocardial infarction (AMI) affects 4% to 14% of patients hospitalized in the intensive care unit (ICU) for non-cardiac disease [1–3] (vs. ≃2 per 1000 admissions for non-cardiac causes in general wards [4]) and is independently associated with increased mortality [1]. In this setting, myocardial ischemia caused by obstructive coronary artery disease (OCAD), designated as a type 1 myocardial infarction [5], is one of the main underlying mechanisms [6]. Decisions regarding use of reperfusion therapy by percutaneous coronary intervention (PCI) in association with dual antiplatelet therapy [6, 7] should be based on the balance of benefit versus risk for each patient, including the risks of ischemia, bleeding, and death [8]. In these patients, inflammation, a prothrombotic state, and endothelial dysfunction [9–13] may increase the ischemic risk [14–16], while platelet disorders [17] and coagulopathy [18] may increase the risk of bleeding [19]. Thus, assessment of the clinical risk is challenging [20], which may explain, in part, why half of these patients do not receive standard of care management as defined in European guidelines [6, 7]. We therefore conducted a multicenter retrospective study in patients with AMI with OCAD during an ICU stay for non-cardiac disease, to assess the incidence and predictive factors of poor outcome, using a composite endpoint of severe ischemic event (AMI recurrence, ischemic stroke), major bleeding, or all-cause death.

## Material and methods

### Selection of patients

In this multicenter retrospective study in three medical ICUs and one mixed medical-surgical ICU of four university teaching hospitals (Paris, France), all consecutive adult patients who had an AMI with OCAD during their ICU stay between January 2012 and December 2018 were considered for inclusion. Diagnosis of AMI with OCAD was based on (i) an elevated cardiac troponin value greater than the 99th percentile of the upper reference level with an increase and/or decrease in troponin values (analytical characteristics of cardiac troponin assays in the different centers are provided in Additional file 1: Table S1) with at least one sign suggestive of myocardial ischemia (typical chest pain, electrocardiogram [ECG] changes, or significant left ventricular (LV) systolic dysfunction [LV ejection fraction [LVEF] ≤ 45%] on echocardiography [2]) [2], and (ii) a coronary angiography showing OCAD (detailed definition in Additional file 1: Table S2) [5]. Exclusion criteria were cardiac disease (myocardial infarction, myocarditis, cardiac rhythm disorders, cardiogenic shock, or cardiogenic pulmonary edema) as a principal diagnosis on ICU admission and cardiac surgery, PCI, or coronary artery bypass grafting within the month prior to ICU admission.

ICU patients who had coronary angiography were identified by the investigator of each participating center, either from hospital medical reports, using the function “research for file in which the word ‘coronary angiography’ occurs” of Microsoft Windows®, or through a search using the following International Classification of Diseases (10th revision) codes: I21 (‘AMI’), I22 (‘subsequent AMI’), R93.1 (‘abnormal findings on diagnostic imaging of heart and coronary circulation’), Z13.6 (‘special screening examination for cardiovascular disorders’), I25.1 (‘atherosclerotic heart disease’). The medical records (including clinical observations, hospitalization reports, as well as electrocardiogram, biological and radiological examinations) of each identified patient were reviewed by the investigators to first verify the inclusion criteria, and second to collect the data. The presence of typical chest pain up to 7 days prior to the day of troponin elevation was noted (it was considered not to be present in patients under sedation). All ECGs performed on the day of troponin elevation were systematically reviewed. This observational, non-interventional analysis of medical records was approved by the Institutional Review Board of the French Society of Intensive Care (CE SRLF 20-76). As per French law, no informed consent was required for this type of study.

### Collection of data

Patient demographics, past medical history, prior antithrombotic treatments, admission category (medical, scheduled surgery, emergency surgery), the principal diagnosis, and the Simplified Acute Physiologic Score II (SAPSII [21]) were recorded on ICU admission. Sepsis and septic shock were defined in accordance with the Sepsis-3 definition [22], and sites of infection were recorded. At the onset of the AMI, the thrombolysis in myocardial infarction (TIMI) risk score [23], the sequential organ failure assessment (SOFA) score [24], the presence of cardiogenic shock, routine blood test results, and details regarding the management of organ failure and of the AMI were collected. Early mechanical reperfusion therapy was defined as coronary reperfusion by PCI or coronary artery bypass graft (CABG) surgery within the first 24 h for ST elevation AMI (STEMI), and within 72 h for non-ST elevation AMI [6, 7].

### Outcomes

The primary endpoint was the occurrence of a composite clinical outcome, including a severe ischemic event (AMI recurrence, stroke), major bleeding (according to the Bleeding Academic Research Consortium, BARC [25]), or death from any cause from the day of AMI (day-1) until hospital discharge. Secondary outcomes were the occurrence of individual components of the primary outcome from the day of AMI until hospital discharge (Detailed definitions in Additional file 1: Table S2) and were not mutually exclusive.

### Statistical analysis

Categorical variables are given as numbers (percentages) and quantitative variables as medians (interquartile ranges [IQR]). Associations with the composite primary outcome were tested using standard Cox models. Potential predictive factors were chosen according to their clinical relevance and their statistical significance (p ≤ 0.05) in the primary outcome univariate analysis. To avoid overfitting, we considered that we could enter a maximum of four variables in our primary outcome model (in view of the 48 events observed) [26]. A multivariable model was built for the primary outcome only, as the number of events was judged too low to avoid overfitting for the other outcomes. Associations with secondary outcomes (individual components of the composite primary outcome) were tested using univariate cause-specific Fine-Gray models for the first occurrence of severe ischemic event or the first occurrence of major bleeding event (accounting for the competing risk of death), and by standard Cox models for all-cause in-hospital mortality. The proportional hazard assumption was assessed through inspection of Schoenfeld residuals. Sensitivity analyses accounting for time and center effect were performed. No power calculation was necessary in view of the methodology used. Hazard ratios (HRs) were estimated and are reported with their 95% confidence intervals (CIs). The level of significance was set a priori at 0.05. Statistical analyses were performed with R software 3.6.0 version for Mac (Foundation for statistical Computing, Vienna, Austria).

## Results

### Population characteristics

**Fig. 1:**
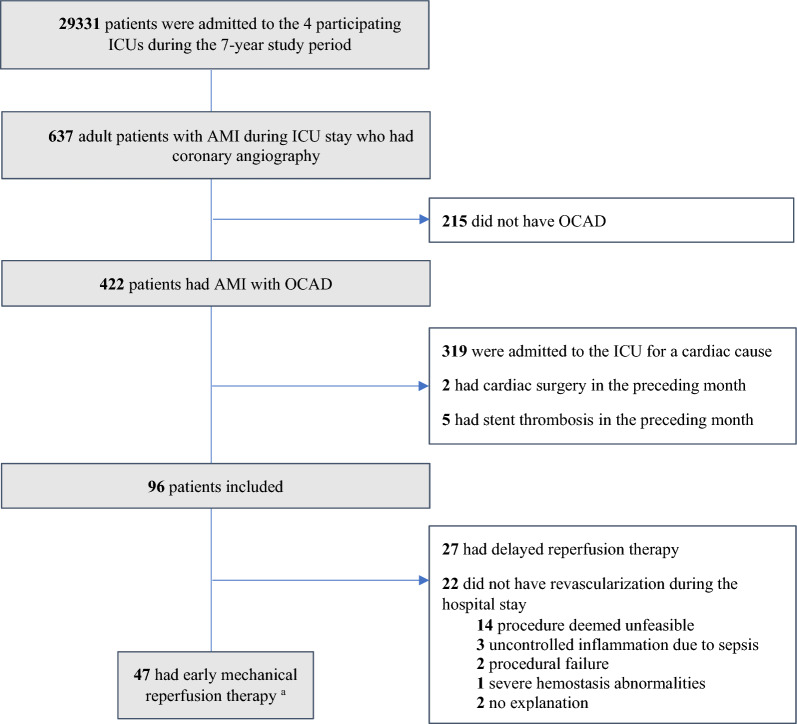
Study flowchart. AMI: acute myocardial infarction; ICU: intensive care unit. ^a^ Within the first 24 h for ST elevation AMI and 72 h for non-ST elevation AMI [6, 7]

During the 7-year study period, 637 adult patients with an AMI had coronary angiography (2.2% of the patients admitted, Fig. 1). Among this population, 96 patients (median age of 69 years [60–78]; 74 men and 22 women, 59 [61%] admitted for sepsis/septic shock) met the study inclusion criteria (Fig. 1, main characteristics in Table 1). The AMI occurred on the day of ICU admission in 83% of the patients (min–max: 0–10 days). On the day of the AMI, the median TIMI and SOFA scores were 4 [3–5] and 8 [3–11], respectively, and the median cardiac troponin peak value was 131 (44–303) times the upper reference limit (URL). Typical chest pain, ECG modifications, and significant LV systolic dysfunction were observed in 28 (29%), 87 (91%), and 50 (52%) patients, respectively (Table 2). Coronary angiography was performed a median (IQR) of 1 (0–6) day after AMI and revealed one vessel-disease, two vessel-disease, and three vessel-disease in 52 (54%), 18 (19%), and 20 (21%) patients, respectively (left main artery, n = 8; left anterior descending artery, n = 54; left circumflex artery, n = 44; right coronary artery, n = 52).

**Table 1:** Baseline characteristics according to occurrence of composite outcome components

Variable	Total (n = 96)	Severe ischemic event^a^		Major bleeding event^b^		Death	
		Yes (*n* = 17)	No (*n* = 79)	Yes (*n* = 26)	No (*n* = 70)	Yes (*n* = 26)	No (*n* = 70)
Age, years	69 [60–78]	68 [65–76]	69 [60–79]	68 [60–76]	69 [59–79]	77 [65–79]	69 [59–77]
Female	22 (23)	2 (12)	20 (25)	4 (15)	18 (26)	7 (27)	15 (21)
BMI (MD, n = 9)	25 [22–29]	26 [23–30]	24 [22–29]	24 [22–28]	25 [22–29]	25 [23–29]	25 [22–29]
Smoker (MD, n = 5)	64 (70)	13 (76)	51 (65)	17 (65)	47 (67)	15 (58)	49 (70)
Past medical history							
Diabetes mellitus	39 (41)	7 (41)	32 (40)	10 (38)	29 (41)	11 (42)	28 (40)
Dyslipidemia	50 (52)	8 (47)	42 (53)	11 (42)	39 (56)	11 (42)	39 (56)
Arterial hypertension	65 (68)	13 (76)	52 (66)	20 (77)	45 (64)	20 (77)	45 (64)
Coronary artery disease	40 (42)	6 (35)	34 (43)	9 (35)	31 (44)	12 (46)	28 (40)
Vascular disease	34 (35)	6 (35)	28 (35)	8 (31)	26 (37)	11 (42)	23 (33)
Chronic kidney failure	14 (15)	4 (23)	10 (13)	6 (23)	8 (11)	2 (8)	12 (17)
Neoplasia	9 (9)	2 (12)	7 (9)	3 (11)	6 (9)	2 (8)	7 (10)
Gastric ulcer	9 (9)	1 (6)	8 (10)	2 (8)	7 (10)	2 (8)	7 (10)
Inflammatory disease	13 (14)	1 (6)	12 (15)	6 (23)	7 (10)	1 (4)	12 (17)
Prior aspirin use	52 (54)	9 (53)	43 (54)	16 (52)	36 (55)	14 (54)	38 (54)
Prior anticoagulation use	12 (13)	2 (12)	10 (13)	3 (10)	9 (14)	3 (11)	9 (13)
Admission category							
Medical	84 (88)	13 (76)	71 (90)	26 (84)	58 (89)	25 (96)	59 (84)
Scheduled surgery	5 (5)	1 (6)	4 (5)	2 (6)	3 (4)	0	5 (7)
Emergency surgery	7 (7)	3 (18)	4 (5)	3 (10)	4 (6)	1 (4)	6 (9)
Intensive care unit diagnosis^c^							
Sepsis or septic shock^d^	59 (61)	11 (65)	48 (61)	15 (58)	44 (63)	19 (73)	40 (57)
Respiratory disease	43 (45)	7 (41)	36 (46)	9 (35)	34 (49)	9 (35)	34 (49)
Urologic disease	22 (23)	2 (12)	20 (25)	6 (23)	16 (23)	6 (23)	16 (23)
Abdominal disease^e^	12 (12)	4 (23)	8 (10)	8 (31)*	4 (6)	3 (11)	9 (13)
Neurologic disease	2 (2)	1 (6)	1 (1)	0	2 (3)	0	2 (3)
Toxic	4 (4)	0	4 (5)	0	4 (6)	1 (4)	3 (4)
Other acute conditions	18 (19)	4	14 (18)	3 (12)	9 (13)	7 (27)*	5 (7)
SAPS II^f^	46 [33–61]	52 [46–65]	45 [32–56]	45 [33–58]	47 [32–62]	64 [48–82] *	41.5 [31–51]

Continuous variables are medians [25th–75th percentile]. Categorical variables are numbers (percentages)

Abbreviations: BMI, body mass index; MD, missing data; SAPS II, simplified acute physiology score II

^a ^Composite of acute myocardial infarction recurrence and stroke

^b ^According to the Bleeding Academic Research Consortium [25]

^c ^At ICU admission. A patient may have more than one intensive care diagnosis

^d ^Site of sepsis: 35 pulmonary, 11 urological, 4 digestive, 3 bacteremia, 2 endocarditis, 2 catheter-related infections, 1 erysipelas, 1 surgical site infection

^e ^5 digestive surgeries, 5 gastrointestinal bleeding, 1 acute pancreatitis, 1 mesenteric ischemia

^f ^SAPS II score range from 0 (lowest) to 163 (highest)

* Univariate comparison with p < 0.05

**Table 2:** Symptoms, laboratory and electrocardiogram findings, and sequential organ failure assessment scores, on the day of the acute myocardial infarction according to occurrence of composite outcome components

Variable	Total (n = 96)	Severe ischemic event^a^		Major bleeding event^b^		All cause death	
		Yes (n = 17)	No (n = 79)	Yes (n = 26)	No (n = 70)	Yes (n = 26)	No (n = 70)
Typical chest pain	28 (29)	5 (29)	23 (29)	9 (35)	19 (27)	4 (15)	24 (34)
Cardiogenic shock	24 (25)	4 (23)	20 (25)	4 (15)	20 (29)	13 (50)*	11 (16)*
Electrocardiogram changes	87 (91)	17 (100)	70 (89)	23 (88)	64 (91)	65 (93)	22 (85)
STEMI	35 (37)	5 (29)	30 (38)	6 (23)	29 (41)	8 (31)	27 (39)
ST segment elevation	31 (32)	4 (23)	27 (34)	6 (23)	25 (36)	6 (23)	25 (36)
New onset left bundle branch block	5 (5)	2 (12)	3 (4)	1 (4)	4 (6)	2 (8)	3 (4)
ST segment depression	37 (39)	9 (53)	28 (35)	12 (46)	25 (36)	7 (27)	30 (43)
T wave inversion	39 (41)	10 (59)	29 (37)	11 (42)	28 (40)	8 (31)	31 (44)
Q wave	14 (15)	1 (6)	13 (16)	2 (8)	12 (17)	3 (11)	11 (16)
Laboratory findings							
Cardiac troponin, times the URL^c^	62 [15–190]	62 [24–124]	61 [12–194]	53 [10–203]	64 [16–173]	61 [16–161]	80 [12–215]
Cardiac troponin peak, times the URL^c^	131 [44–303]	173 [50–236]	126 [39–319]	163 [46–301]	125 [43–301]	131 [49–258]	138 [17–394]
Hematocrit, % (MD = 6)	34 [28–40]	30 [27–39]	35 [28–41]	28 [25–34]*	35 [30–41]	32 [28–35]	35 [28–41]
Platelets, 10^3^/mm^3^ (MD = 1)	221 [175–291]	204 [140–274]	224 [178–303]	213 [143–284]	225 [181–310]	195 [127–265]*	224 [191–310]
Plasma creatinine, µmol/L ( MD = 2)	130 [91–196]	146 [96–207]	129 [86–189]	134 [89–296]*	127 [92–174]	144 [101–235]	120 [90–186]
PH (MD = 3)	7.34 [7.22–7.42]	7.37 [7.29–7.41]	7.34 [7.20–7.42]	7.36 [7.31–7.42]	7.33 [7.20–7.42]	7.35 [7.22–7.41]	7.34 [7.21–7.42]
PaO2/FiO2 ratio (MD = 5)	222 [149–337]	189 [157–405]	228 [148–330]	203 [133–305]	223 [151–337]	199 [148–247]	234 [152–349]
Lactate, mmol/L (MD = 6)	2.5 [1.5–4.6]	2.1 [1.5–4.5]	2.6 [1.5–4.6]	1.9 [1.4–3.8]	3.0 [1.6–5.2]	3.1 [1.6–7.4]	2.4 [1.4–4.1]
Left ventricular systolic dysfunction, (LVEF ≤ 45%), No. (%) (MD = 26)	50 (70)	9 (53)	41 (52)	12 (46)	38 (54)	39 (56)	11 (42)
TIMI risk score^d^	4 [3–5]	4 [3–5]	4 [3–5]	4 [3–5]	4 [3–5]	4 [3–5]	4 [3–5]
SOFA global score	8 [3–11]	9 [4–12]	7 [3–11]	6 [4–11]	8 [3–11]	10 [8–12]*	5 [2–10]
SOFA Cardiovascular score	2 [0–4]	4 [0–4]	1 [0–4]	1 [0–4]	4 [0–4]	4 [4–4] *	1 [0–4]

Continuous variables are medians [25th–75th percentile]. Categorical variables are numbers (percentages)

Abbreviations: LVEF: left ventricular ejection fraction; MD: missed data; SOFA: sequential organ failure assessment; STEMI: ST elevation myocardial infarction; TIMI: Thrombolysis in Myocardial Infarction; URL, upper reference limit

^a ^Composite of acute myocardial infarction recurrence and stroke

^b ^According to the Bleeding Academic Research Consortium [25]

^c ^The URL (different for each center) corresponding to the 99th percentile value for the overall population. More details in Additional file 1, Table S1

^d ^Derived in patients with non-ST-segment elevation myocardial infarction to predict 14-day outcomes, including all-cause mortality, new or recurrent myocardial infarction or severe recurrent ischaemia requiring urgent revascularization [23]

* Univariate comparison with p < 0.05

### Management

On the day of the AMI, antiplatelet therapy, dual antiplatelet therapy, or therapeutic anticoagulation was administered in 95 (99%), 61 (63%), and 68 (71%) patients, respectively (Table 3). Early mechanical reperfusion therapy was performed in 47 (49%) patients (Fig. 1), including PCI in 45 patients (drug-eluting stent, n = 31; bare metal stent, n = 13; missing data, n = 1) and CABG in two patients. Delayed mechanical reperfusion therapy was performed in 27 (28%) patients, including PCI in 21 patients (drug-eluting stent, n = 14; bare metal stent, n = 7) and CABG in six patients. The reasons for delayed mechanical reperfusion were uncontrolled sepsis (n = 16), hemodynamic instability (n = 2), active bleeding (n = 3), triple vessel disease (n = 4), complex procedure (n = 1), and unknown (n = 1). Organ failure management on the day of AMI onset included catecholamines in 48 (51%) patients and invasive mechanical ventilation in 56 (58%, Table 3).

**Table 3:** Management of myocardial infarction and of organ dysfunction on the day of the myocardial infarction according to occurrence of composite outcome components

Variable	Total (n = 96)	Severe ischemic event^a^		Major bleeding event^b^		Death	
		Yes (*n* = 17)	No (*n* = 79)	Yes (*n* = 26)	No (*n* = 70)	Yes (*n* = 26)	No (*n* = 70)
Myocardial infarction management							
Antiplatelet therapy	95 (99)	17 (100)	78 (99)	25 (96)	70 (100)	26 (100)	69 (99)
Dual antiplatelet therapy	61 (64)	10 (59)	51 (65)	11 (42) *	50 (71)	16 (61)	45 (64)
Therapeutic anticoagulation (MD = 1)	68 (71)	11 (65)	57 (72)	18 (69)	50 (71)	18 (69)	50 (71)
Early mechanical reperfusion therapy^c^^, d^	47 (49)	9 (53)	38 (48)	12 (46)	35 (50)	13 (50)	34 (49)
Organ dysfunction management							
Catecholamines	48 (51)	10 (59)	38 (48)	11 (42)	37 (53)	21 (81) *	27 (39)
Invasive mechanical ventilation	56 (58)	14 (82)	42 (53)	17 (65)	39 (56)	23 (88) *	33 (47)
Renal replacement therapy	9 (9)	3 (18)	6 (8)	5 (19)	4 (6)	2 (8)	7 (10)
VA-ECMO	3 (3)	2 (12)	1 (1)	1 (4)	2 (3)	3 (11) *	0

Variables are numbers (percentages)

Abbreviations: MD: missing data; VA-ECMO: veno-arterial extracorporeal membrane oxygenation

^a ^Composite of acute myocardial infarction recurrence and stroke

^b^According to the Bleeding Academic Research Consortium [25]

^c ^Percutaneous coronary intervention, n = 45; coronary artery bypass graft, n = 2

^d ^Within the first 24 h for ST elevation acute myocardial infarction, and within 72 h for non-ST elevation acute myocardial infarction

*Univariate comparison with p < 0.05

### Composite clinical outcome and associated patient factors

The composite clinical outcome occurred in 48 (50%) patients (cumulative incidence curve in Additional file 1: Fig. S1). Patients with a composite clinical outcome more frequently had a history of arterial hypertension (79% vs. 56%, p = 0.03) and diagnosis of abdominal disease on ICU admission (23% vs 2%, p = 0.005), higher SAPS II (50 [43–73] vs. 40 [27–49], p < 0.001), higher SOFA global and SOFA cardiovascular scores (respectively, 9 [5–11] vs. 4 [2–10], p = 0.004; 4 [0–4] vs. 0 [0–4], p = 0.016), and lower hematocrit levels (30 [26–35]% vs. 37 [32–43]%, p = 0.001) (Additional file 1, Tables S3 and S4). The occurrence of the composite clinical outcome was similar in patients with and without sepsis/septic shock (Additional file 1: Table S5). AMI management in terms of antithrombotic medication and early mechanical reperfusion was not significantly different between the groups with and without the composite clinical outcome, but a greater proportion of patients with the composite outcome received catecholamines and invasive mechanical ventilation on the day of AMI (Additional file 1: Table S6).

A history of arterial hypertension, diagnosis of abdominal disease on ICU admission, SOFA score, and peak cardiac troponin were entered in the multivariable model, and history of arterial hypertension (HR 2.05, 95% CI 1.01–4.16, p = 0.047) and high SOFA score (HR 1.07, 95% CI 1.00–1.15, p = 0.042) were identified as independently associated with an increased risk of the composite outcome (Table 4). There was no significant time (Additional file 1: Table S7) or center (Additional file 1: Table S8) effect on the occurrence of the composite outcome.

**Table 4:** Univariate and multivariable analyses of factors associated with in-hospital outcome ^a^

Variable	Univariate analysis		Multivariable model^b^	
	HR (95% CI)	p value	HR (95% CI)	p value
History of arterial hypertension	2.01 (1.00–4.03)	0.05	2.05 (1.01–4.16)	0.047
Abdominal disease	2.35 (1.19–4.64)	0.014	1.80 (0.88–3.68)	0.11
SOFA global	1.09 (1.02–1.16)	0.014	1.07 (1.00–1.15)	0.042
Cardiac troponin peak, times the URL^c, d^	1.08 (0.90–1.28)	0.41	1.04 (0.87–2.25)	0.67

Abbreviations: HR: hazard ratio; SOFA: Sequential Organ Failure Assessment; URL: upper limit of reference

^a ^Composite of severe ischemic event, major bleeding event, or all-cause death

^b ^Adjusted on history of arterial hypertension, abdominal disease, SOFA global, and cardiac troponin I peak times the URL

^c ^Log10 transformation of cardiac troponin peak to normalize distribution

^d ^The URL (different for each center) corresponding to the 99th percentile value for the overall population. More details in Additional file 1: Table S1

### Components of the composite clinical outcome

A severe ischemic event occurred in 17 (18%) patients (median 5 [3–7] days from AMI onset), including 9 recurrent AMIs and 8 strokes. One severe ischemic event (recurrent AMI related to early stent thrombosis) was fatal. Baseline clinical (Table 1) and AMI characteristics (Table 2) were similar in patients with and without a severe ischemic event.

Major bleeding occurred in 26 (27%) patients (median 4 [3–13] days from AMI onset) including 40 major extracranial bleeding events (23 gastrointestinal, 1 hemoptysis, 1 epistaxis, 1 thigh hematoma, 5 urologic, 1 pericardial effusion, 8 surgical site) and 1 intracranial bleeding event; no episode of major bleeding was fatal. A blood transfusion (median number of red blood cell units 2 [1–5]) was required for 34 of the major bleeding events. The 41 major bleeding events were classified as follows: BARC 3a, n = 24; BARC 3b, n = 14; BARC 3c, n = 1; BARC 4, n = 2. Patients with a major bleeding event had more frequently been admitted to the ICU for abdominal disease, and had a lower hematocrit and less often received dual antiplatelet therapy on the day of AMI. Six patients had both a severe ischemic event and a major bleeding event.

Twenty-six (27%) patients died in the hospital. The causes of death were refractory cardiogenic shock (n = 7), cardiac arrest of cardiogenic origin (or suspected) (n = 5), multiple organ failure (n = 7), and end-of-life decision (n = 7). SAPS II, SOFA global, and SOFA cardiovascular scores were higher in non-survivors.

The occurrence of each component of the composite clinical outcome was similar in patients with and without sepsis or septic shock (Additional file 1: Table S5).

## Discussion

In this retrospective multicenter study in patients with AMI with OCAD during an ICU stay for non-cardiac disease, the incidence of the composite in-hospital outcome, including severe ischemic event (18%), major bleeding (27%), and mortality (27%), was high (50%). A history of arterial hypertension and a high SOFA score were independently associated with a risk of poor outcome.

### Ischemic risk

Our results confirm that risk of ischemia is a major concern in patients with an AMI during an ICU stay for non-cardiac disease (majority with sepsis). Smilowitz et al. reported that AMI was independently associated with increased mortality in a retrospective analysis of a large nationwide cohort of patients with sepsis [1]. The ischemic risk in this setting appears to be greater than that reported in patients with AMI in cardiology wards (< 5%) [27]. The pathophysiological mechanisms behind the increased ischemic risk are complex in this context. Myocardial infarction may be a marker of the severity of non-cardiac disease, such as septic shock, which itself is associated with a high thrombotic risk because of hemodynamic collapse, sepsis-induced coagulopathy with deregulated immunothrombosis, and endothelial dysfunction [28–30]. Several infectious agents and inflammatory diseases are associated with an increased risk of AMI, probably related to the overall burden of systemic inflammation [5, 31–33] that could lead to coronary plaque instability and thrombus formation. In an observational study, Del Pace et al. showed that occurrence of an infectious or inflammatory event may facilitate the development of coronary stent thrombosis [33]. In addition, tachycardia and blood pressure changes in critically ill patients can precipitate plaque rupture and coronary thrombosis [34]. Furthermore, the bioavailability of enteral drugs, such as antiplatelet agents, can be significantly altered in critically ill patients, leading to an increased risk of thrombosis [35, 36]. Finally, early invasive reperfusion was not performed in half our patients, leading to a potential increase in the risk of severe ischemic complications.

### Bleeding risk

Episodes of major bleeding were also frequent, occurring in 27% of our patients. In medical ICU patients, Strauss et al. reported a similar incidence of major bleeding (20%) [19]. Similar to ischemic risk, bleeding risk in this setting appears to be greater than that reported in patients with AMI in cardiology wards (≃ 5%) [37]. The dysregulated infection-inflammation immune response may produce antithrombotic states with thrombocytopenia, decreased clotting factors, and increased fibrinolysis, which predispose to bleeding complications [38–41]. Several studies have developed predictive instruments for the estimation of bleeding risk in patients with AMI in cardiology wards [42]. Subherwal et al. reported that a lower baseline hematocrit was an independent predictor of bleeding events [42]; similarly, in our univariate analysis, patients with bleeding events had lower baseline hematocrit values. We found a counter-intuitive association between dual antiplatelet administration and a lower risk of bleeding events. This may be due to indication bias, or to a high alpha risk given the large number of statistical comparisons in this exploratory observational study.

### Prediction of the composite clinical outcome

Our results are consistent with those from several studies in cardiology patients, which have reported that a history of arterial hypertension is associated with ischemic [23, 43] and bleeding [44] risk. However, no other factors commonly used to stratify cardiovascular risk in cardiology patients [43, 45], such as TIMI risk score, ECG abnormalities and baseline cardiovascular characteristics, were predictive of clinical outcome in our cohort. Thus, the approach used to estimate this risk in cardiology patients [43, 45] may not be relevant in patients admitted to the ICU for a non-cardiac condition. Several authors have reported that invasive reperfusion therapy is performed in only 30–50% of critically ill patients with AMI during an ICU stay for a non-cardiac cause [3, 36], similar to our findings. These observations suggest there is an urgent need for bedside risk stratification tools to determine which patients may benefit most from antithrombotic medications and early invasive reperfusion strategy.

### Limitations

Our study has several limitations. First, this was a retrospective study with inherently associated bias, some missing data, and possible associated errors in data abstraction. Second, all our statistical results should be interpreted with caution in this exploratory retrospective study because of (i) the large number of statistical comparisons and the not adjusted 95% confidence intervals and p-values for multiple testing resulting in high alpha risk, and (ii) the relatively small number of patients limited power in all analyses. Third, severe ischemic events (or major bleeding events) may have been more relevant as a primary outcome, but we did not consider this option, because of their low incidence. Instead, we used a composite outcome that reflects the net clinical benefit of antithrombotic medication and invasive reperfusion strategy. Fourth, we did not assess the relationship between AMI and the occurrence of the composite outcome. Fifth, the study was observational, leading to potential indication biases. Specific treatments for AMI, including antithrombotic therapy and reperfusion therapy, may have influenced the occurrence of adverse events. Sixth, patients without coronary angiography to confirm AMI were not included, leading to potential selection bias. Indeed, many coronary angiographies are delayed or not performed in the acute phase of septic shock because of fear of stent thrombosis due to the pro-thrombotic state of the patients. Seventh, as the study was conducted in France, our findings may not be applicable elsewhere. Finally, the last patient was included in December 2018. However, to the best of our knowledge, no trial results or guidelines have been published since then that could have modified the usual management of these patients.

## Conclusion

Patients having an AMI with OCAD during an ICU stay for non-cardiac disease are at high risk of poor clinical outcome, including development of severe ischemic events or major bleeding, and death. A history of arterial hypertension and a high SOFA score at the time of the AMI were the only factors associated with occurrence of the composite outcome, albeit the relatively small sample size. Further studies are needed to determine how to better stratify bedside cardiovascular risk, a preliminary requirement for establishing an appropriate anti-thrombotic and coronary reperfusion strategy in this context.

### Supplementary Information

**Additional file 1: Table S1.** Analytical characteristics of cardiac troponin assays in the different centers. **Table S2.** Definitions of acute myocardial infarction, severe ischemic events, and major bleeding events. **Table S3.** Baseline patient characteristics according to occurrence of the composite outcome. **Table S4.** Symptoms, laboratory and electrocardiogram findings, and sequential organ failure assessment (SOFA) scores, on the day of the acute myocardial infarction according to occurrence of the composite outcome. **Table S5.** Outcomes according to the intensive care unit diagnosis of sepsis/ septic shock. **Table S6.** Management of myocardial infarction and of organ dysfunction on the day of the acute myocardial infarction according to the occurrence of the composite outcome. **Table S7.** Sensitivity analysis of factors associated with occurrence of composite outcomes accounting for time effect. **Table S8.** Sensitivity analysis of factors associated with occurrence of composite outcome accounting for center effect: mixed effects Cox model with center as random effect. **Figure S1.** Composite Clinical Outcome. (A) Cumulative composite clinical outcome curve (including any severe ischemic event, major bleeding and all-cause death) from the day of AMI until hospital discharge. (B) Cumulative mortality curve from the day of AMI until hospital discharge. (C) Cumulative severe ischemic events curve from the day of AMI until hospital discharge. (D) Cumulative major bleedings curve from the day of AMI until hospital discharge.

## Data Availability

The datasets used and analyzed during the current study are available from the corresponding author on reasonable request.

## Abbreviations

AMI: Acute myocardial infarction
ECG: Electrocardiogram
ICU: Intensive care unit
IQR: Interquartile range
LVEF: Left ventricular ejection fraction
OCAD: Obstructive coronary artery disease
PCI: Percutaneous coronary intervention
SAPS II: Simplified Acute Physiology Score II
SOFA: Sequential Organ Failure Assessment
STEMI: ST elevation myocardial infarction
URL: Upper reference limit
