# Supplementary material for: In-hospital outcomes after acute myocardial infarction with obstructive coronary artery disease in critically ill patients hospitalized for non-cardiac disease
Source: Ann Intensive Care. 2023 Sep 19;13:87. doi: 10.1186/s13613-023-01188-9 (PMC10509106; doi:10.1186/s13613-023-01188-9)
Supplement: Supplementary file 1 — Additional file 1: Table S1. Analytical characteristics of cardiac troponin assays in the different centers. Table S2. Definitions of acute myocardial infarction, severe ischemic events, and major bleeding events. Table S3. Baseline patient characteristics according to occurrence of the composite outcome. Table S4. Symptoms, laboratory and electrocardiogram findings, and sequential organ failure assessment (SOFA) scores, on the day of the acute myocardial infarction according to occurrence of the composite outcome. Table S5. Outcomes according to the intensive care unit diagnosis of sepsis/ septic shock. Table S6. Management of myocardial infarction and of organ dysfunction on the day of the acute myocardial infarction according to the occurrence of the composite outcome. Table S7. Sensitivity analysis of factors associated with occurrence of composite outcomes accounting for time effect. Table S8. Sensitivity analysis of factors associated with occurrence of composite outcome accounting for center effect: mixed effects Cox model with center as random effect. Figure S1. Composite Clinical Outcome. (A) Cumulative composite clinical outcome curve (including any severe ischemic event, major bleeding and all-cause death) from the day of AMI until hospital discharge. (B) Cumulative mortality curve from the day of AMI until hospital discharge. (C) Cumulative severe ischemic events curve from the day of AMI until hospital discharge. (D) Cumulative major bleedings curve from the day of AMI until hospital discharge. [file 13613_2023_1188_MOESM1_ESM.docx]

**ADDITIONAL FILE**

[Table S1. Analytical characteristics of cardiac troponin assays in the different centers 2](#_Toc140064042)

[Table S2. Definitions of acute myocardial infarction, severe ischemic events, and major bleeding events 3](#_Toc140064043)

[Table S3. Baseline patient characteristics according to occurrence of the composite outcome ^a^. 5](#_Toc140064044)

[Table S4. Symptoms, laboratory and electrocardiogram findings, and sequential organ failure assessment (SOFA) scores, on the day of the acute myocardial infarction according to occurrence of the composite outcome ^a^ 6](#_Toc140064045)

[Table S5. Outcomes according to the intensive care unit diagnosis of sepsis/ septic shock ^a^. 7](#_Toc140064046)

[Table S6. Management of myocardial infarction and of organ dysfunction on the day of the acute myocardial infarction according to the occurrence of the composite outcome ^a^ 8](#_Toc140064047)

[Table S7. Sensitivity analysis of factors associated with occurrence of composite outcomes ^a^ accounting for time effect 9](#_Toc140064048)

[Table S8. Sensitivity analysis of factors associated with occurrence of composite outcome ^a^ accounting for center effect: mixed effects Cox model with center as random effect 10](#_Toc140064049)

[Figure S1. Composite Clinical Outcome. (A) Cumulative composite clinical outcome curve (including any severe ischemic event, major bleeding and all-cause death) from the day of AMI until hospital discharge. (B) Cumulative mortality curve from the day of AMI until hospital discharge. (C) Cumulative severe ischemic events curve from the day of AMI until hospital discharge. (D) Cumulative major bleedings curve from the day of AMI until hospital discharge. 11](#_Toc140064050)

[References 12](#_Toc140064051)

# Table S1. Analytical characteristics of cardiac troponin assays in the different centers

| **Center** | **Company, platform, assay** | **Limit of detection** | **99^th^ percentile URL** |
| --- | --- | --- | --- |
| Center 1 before December 2014 | Abbott Architect cTnI | 0.01 µg/L | 0.03 µg/L |
| Center 1 from December 2014 | Abbott Architect hs-cTnI | 1.1 ng/L | 26 ng/L |
| Center 2 | Roche hs-cTnT | 3 ng/L | 14 ng/L |
| Center 3 | Beckman Coulter Access AccuTnI+3 | 0.01 µg/L | 0.1 µg/L |
| Center 4 | Roche hs-cTnT | 3 ng/L | 14 ng/L |
| Abbreviations: cTnI, cardiac troponin I; hs-cTnI, high-sensitivity cardiac troponin I; hs-cTnT, high-sensitivity cardiac troponin T; URL, upper reference limit | | | |

| Table S2. Definitions of acute myocardial infarction, severe ischemic events, and major bleeding events | |
| --- | --- |
| **Variable** | **definition** |
| **Acute myocardial infarction** | Acute myocardial injury with clinical evidence of acute myocardial ischemia (based on the fourth universal definition of myocardial infarction 2018[1] |
| **Presence of:** |  |
| Acute myocardial injury | Detection of a rise and/or fall of cardiac Troponin values with at least one value above  the 99th percentile URL |
| **And at least one of the following:** |  |
| Clinical evidence of acute myocardial ischemia | Symptoms of myocardial ischemia: typical chest pain radiating to upper extremity and/or mandible. ^a^ |
| Electrocardiogram signs of myocardial ischemia | New (or presumed new) ischemic ECG changes (significant ST segment or T wave changes or a new left branch block) or appearance of pathologic Q waves. |
| Imaging evidence of wall motion abnormality | New (or presumed new) significant left ventricular systolic dysfunction (echocardiographic left ventricular ejection fraction (LVEF) ≤ 45%).^b^ LVEF was assessed using the bi-plane Simpson method, or was visually estimated in case of inadequate identification of the endocardium. |
| **Severe ischemic event** | |
| Myocardial infarction recurrence | Myocardial infarction associated with coronary reperfusion procedures. Type IV (a or b) and type V myocardial infarction according to the international definition [1]. |
| Stroke | Focal neurological deficit of abrupt onset, lasting more than 24 hours, for which neuroimaging (CT scan or MRI) excludes any other potential cause, in particular a cerebral hemorrhage (excluding hemorrhagic transformation of an ischemic stroke). |
| **Major bleeding events, according** **to the Bleeding Academic Research Consortium (BARC)** [2] | |
| BARC 3a | - Bleeding associated with a decrease in hemoglobin of 3-5 g/dl or need for blood transfusion. |
| BARC 3b | - Bleeding associated with a decrease in hemoglobin > 5 g/dl - Cardiac tamponade. - Need for surgical control of bleeding (except dental, nasal, skin, hemorrhoid). - Need for vasoactive drugs. |
| BARC 3c | - Intracranial bleeding (except for micro-bleeding or hemorrhagic transformation ; includes intraspinal bleeding). - Intraocular bleeding with vision impairment. |
| BARC 4 | - Bleeding related to coronary artery bypass graft (CABG). - Perioperative intracranial bleeding within 48 hours. - Reoperative after closure of sternotomy to control bleeding. - Transfusion ≥ 5 packed red blood cells within a 48-hour period - Chest tube bleeding ≥ 2 L in 24 hours. |
| BARC 5a | - Probable fatal bleeding: clinical suspicion not confirmed by autopsy or imaging. |
| BARC 5b | - Definitive fatal bleeding: active bleeding or confirmed by autopsy or imaging methods. |
| ^a^ Other non-specific symptoms of myocardial ischemia such as dyspnea, fatigue, or epigastric discomfort were not considered  ^b^ In this pragmatic retrospective study, any significant new (or suspected) left ventricular systolic dysfunction, whether regional (according to the fourth universal definitions of myocardial infarction [3]) or global, was considered imaging evidence of wall motion abnormality. | |

| Table S3. Baseline patient characteristics according to occurrence of the composite outcome ^a^. | | | |
| --- | --- | --- | --- |
| **Variables** | **Composite outcome**  **(*n*=48)** | **No composite outcome**  **(*n*=48)** | ***p*** |
| Age, years | 69 [62–78] | 69 [58–77] | 0.28 |
| Female | 10 (21) | 12 (25) | 0.81 |
| BMI (MD, n=9) | 25 [22–29] | 25 [22–29] | 0.93 |
| Smoker (MD, n=5) | 29 (66) | 35 (74) | 0.51 |
| Past medical history |  |  |  |
| Diabetes mellitus | 19 (40) | 20 (42) | >0.99 |
| Dyslipidemia | 23 (48) | 27 (56) | 0.54 |
| Arterial hypertension | 38 (79) | 27 (56) | 0.03 |
| Coronary artery disease | 20 (42) | 20 (42) | >0.99 |
| Vascular disease | 20 (42) | 14 (29) | 0.29 |
| Chronic kidney failure | 9 (19) | 5 (10) | 0.39 |
| Neoplasia | 5 (10) | 4 (8) | >0.99 |
| Gastric ulcer | 4 (8) | 5 (10) | >0.99 |
| Inflammatory disease | 6 (12) | 7 (15) | >0.99 |
| Prior aspirin use | 28 (58) | 24 (50) | 0.54 |
| Prior anticoagulation use | 5 (10) | 7 (15) | 0.76 |
| Admission category |  |  |  |
| Medical | 40 (83) | 44 (92) | 0.35 |
| Scheduled surgery | 3 (6) | 2 (4) | >0.99 |
| Emergency surgery | 5 (10) | 2 (4) | 0.43 |
| Intensive care unit diagnosis |  |  |  |
| Sepsis or septic shock ^b^ | 30 (62) | 29 (60) | >0.99 |
| Respiratory disease | 16 (33) | 27 (56) | 0.04 |
| Urologic disease | 11 (23) | 11 (23) | >0.99 |
| Abdominal disease | 11 (23) | 1 (2) | 0.005 |
| Neurologic disease | 1 (2) | 1 (2) | >0.99 |
| Toxic | 1 (2) | 3 (6) | 0.61 |
| Other acute conditions | 10 (21) | 8 (17) | 0.79 |
| SAPS II ^c^ | 50 [43–73] | 40 [27–49] | <0.001 |
| Continuous variables are medians [25th–75th percentile]. Categorical variables are numbers (percentages).  Abbreviations: BMI, body mass index; MD, missing data; SAPS II, simplified acute physiology score II.  ^a^ composite of severe ischemic event (acute myocardial infarction recurrence or stroke), major bleeding, or death from any cause  ^b^ Site of sepsis: 35 pulmonary, 11 urological, 4 digestive, 3 bacteremia, 2 endocarditis, 2 catheter-related infections, 1 erysipelas, 1 surgical site infection.  ^c^ SAPS II score range from 0 (lowest) to 163 (highest): disease severity and mortality risk. | | | |

| Table S4. Symptoms, laboratory and electrocardiogram findings, and sequential organ failure assessment (SOFA) scores, on the day of the acute myocardial infarction according to occurrence of the composite outcome ^a^ | | | |
| --- | --- | --- | --- |
| **Variable** | **Composite outcome**  **(n=48)** | **No composite outcome**  **(n=48)** | ***p*** |
| Typical chest pain | 12 (25) | 16 (33) | 0.50 |
| Killip classification | 1 [1–4] | 1 [1–3] | 0.25 |
| Electrocardiogram changes |  |  |  |
| STEMI | 14 (29) | 21 (44) | 0.20 |
| ST segment elevation | 12 (25) | 19 (40) | 0.19 |
| New onset left bundle branch block | 3 (6) | 2 (4) | >0.99 |
| ST segment depression | 19 (40) | 18 (37) | >0.99 |
| T wave inversion | 19 (40) | 20 (42) | >0.99 |
| Q wave | 6 (12) | 8 (17) | 0.77 |
| Laboratory findings |  |  |  |
| Cardiac troponin I, times the URL ^b^ | 69 [15–210] | 61 [15–160] | 0.74 |
| Cardiac troponin peak, times the URL ^b^ | 174 [48–389] | 113 [40–230] | 0.21 |
| Hematocrit, % (MD=6) | 30 [26–35] | 37 [32–43] | 0.001 |
| Platelet count, 10^3^/mm^3^ (MD=1) | 218 [143–276] | 230 [196–324] | 0.08 |
| Plasma creatinine, µmol/L (MD, n=2) | 141 [91–240] | 114 [91–153] | 0.07 |
| pH (MD=3) | 7.35 [7.22–7.42] | 7.33 [7.20–7.39] | 0.44 |
| PaO2/FiO2 ratio (MD=5) | 200 [148–290] | 240 [162–344] | 0.32 |
| Lactate, mmol/L (MD=6) | 2.8 [1.6–5.8] | 2.4 [1.5–3.7] | 0.24 |
| Left ventricular systolic dysfunction (LVEF ≤45%), No. (%) (MD=26) | 24 (75) | 26 (67) | 0.61 |
| TIMI risk score ^c^ | 4 [3–5] | 4 [3–5] | 0.97 |
| SOFA global | 9 [5–11] | 4 [2–10] | 0.004 |
| SOFA cardiovascular | 4 [0–4] | 0 [0–4] | 0.02 |
| Continuous variables are medians [25th–75th percentile]. Categorical variables are numbers (percentages).  Abbreviations: LVEF, left ventricular ejection fraction; MD: missing data; SOFA, sequential organ failure assessment; STEMI, ST elevation myocardial infarction; TIMI: Thrombolysis in Myocardial Infarction; URL, upper reference limit  ^a^ composite of severe ischemic event (acute myocardial infarction recurrence or stroke), major bleeding, or death from any cause  ^b^ The URL (different for each center) corresponding to the 99th percentile value for the overall population. More details in additional file 1 Table S1.  ^c^ Derived in patients with non-ST-segment elevation myocardial infarction to predict 14-day outcomes, including all-cause mortality, new or recurrent myocardial infarction or severe recurrent ischaemia requiring urgent revascularization [4] | | | |

| Table S5. Outcomes according to the intensive care unit diagnosis of sepsis/ septic shock ^a^. | | | |
| --- | --- | --- | --- |
| **Variables** | **Septic patients**  **(*n*=59)** | **Non-septic patients**  **(*n*=37)** | ***p*** |
| Composite outcome | 30 (51) | 18 (49) | >0.99 |
| Severe ischemic event ^b^ | 11 (19) | 6 (16) | 0.98 |
| Major bleeding event ^c^ | 15 (25) | 11 (30) | 0.82 |
| All cause death | 19 (32) | 7 (19) | 0.23 |
| Continuous variables are medians [25th–75th percentile].  ^a^ According to the Sepsis 3 definition [5]  ^b^ Composite of acute myocardial infarction recurrence and stroke.  ^c^ According to the Bleeding Academic Research Consortium (BARC ≥3) [6] | | | |

| Table S6. Management of myocardial infarction and of organ dysfunction on the day of the acute myocardial infarction according to the occurrence of the composite outcome ^a^ | | | |
| --- | --- | --- | --- |
| **Variable** | **Composite outcome (n=48)** | **No composite outcome**  **(n=48)** | ***p*** |
| Myocardial infarction management |  |  |  |
| Antiplatelet therapy | 47 (98) | 48 (100) | >0.99 |
| Dual antiplatelet therapy | 26 (54) | 35 (73) | 0.09 |
| Therapeutic anticoagulation (MD=1) | 31 (66) | 37 (77) | 0.33 |
| Early reperfusion therapy ^b, c^ | 23 (48) | 24 (50) | >0.99 |
| Organ dysfunction management |  |  |  |
| Catecholamines | 30 (62) | 18 (37) | 0.025 |
| Invasive mechanical ventilation | 36 (75) | 20 (42) | 0.002 |
| Renal replacement therapy | 3 (18) | 6 (8) | 0.40 |
| VA ECMO | 3 (6) | 0 | 0.24 |
| Continuous variables are medians (25th–75th percentile). Categorical variables are numbers (percentages).  Abbreviations: MD, missed data; VA ECMO, veno-arterial extracorporeal membrane oxygenation.  ^a^ composite of severe ischemic event (acute myocardial infarction recurrence or stroke), major bleeding, or death from any cause  ^b^ Percutaneous coronary intervention, n=45; coronary artery bypass graft, n=2.  ^c^ Within the first 24 hours for the ST elevation acute myocardial infarction, and within 72 hours for the non-ST elevation acute myocardial infarction | | | |

| Table S7. Sensitivity analysis of factors associated with occurrence of composite outcomes ^a^ accounting for time effect | | |
| --- | --- | --- |
| **Variable** | **HR (95% CI)** | **p value** |
| Arterial hypertension | 2.04 (1.00 – 4.15) | 0.049 |
| Abdominal disease | 1.83 (0.88 – 3.81) | 0.10 |
| SOFA global | 1.07 (1.00 – 1.15) | 0.04 |
| Cardiac troponin peak times the URL ^b, c^ | 1.05 (0.87 – 1.26) | 0.64 |
| Time effect ^c^ | 0.93 (0.49 – 1.75) | 0.82 |
| Abbreviations: SOFA, sequential organ failure assessment; URL, upper reference limit  ^a^ composite of severe ischemic event (acute myocardial infarction recurrence or stroke), major bleeding, or death from any cause  ^b^ Log10 transformation of cardiac troponin peak to normalize distribution.  ^c^ The URL (different for each center) corresponding to the 99th percentile value for the overall population. More details in additional file 1, Table S1. | | |

| Table S8. Sensitivity analysis of factors associated with occurrence of composite outcome ^a^ accounting for center effect: mixed effects Cox model with center as random effect | | |
| --- | --- | --- |
| **Variable** | **HR (95% CI)** | **p Value** |
| Arterial hypertension | 1.9 (0.96 – 3.98) | 0.07 |
| Abdominal disease | 1.79 (0.88 – 3.67) | 0.11 |
| SOFA global | 1.07 (1.00 – 1.15) | 0.04 |
| Cardiac troponin I peak, times the URL ^b, c^ | 1.03 (0.86 – 1.23) | 0.73 |
| Abbreviations: SOFA, sequential organ failure assessment; URL, upper reference limit  ^a^ composite of severe ischemic event (acute myocardial infarction recurrence or stroke), major bleeding, or death from any cause  ^b^ Log10 transformation of cardiac troponin peak to normalize distribution.  ^c^ The URL (different for each center) corresponding to the 99th percentile value for the overall population. More details in additional file 1, Table S1 | | |

# Figure S1. Composite Clinical Outcome. (A) Cumulative composite clinical outcome curve (including any severe ischemic event, major bleeding and all-cause death) from the day of AMI until hospital discharge. (B) Cumulative mortality curve from the day of AMI until hospital discharge. (C) Cumulative severe ischemic events curve from the day of AMI until hospital discharge. (D) Cumulative major bleedings curve from the day of AMI until hospital discharge.


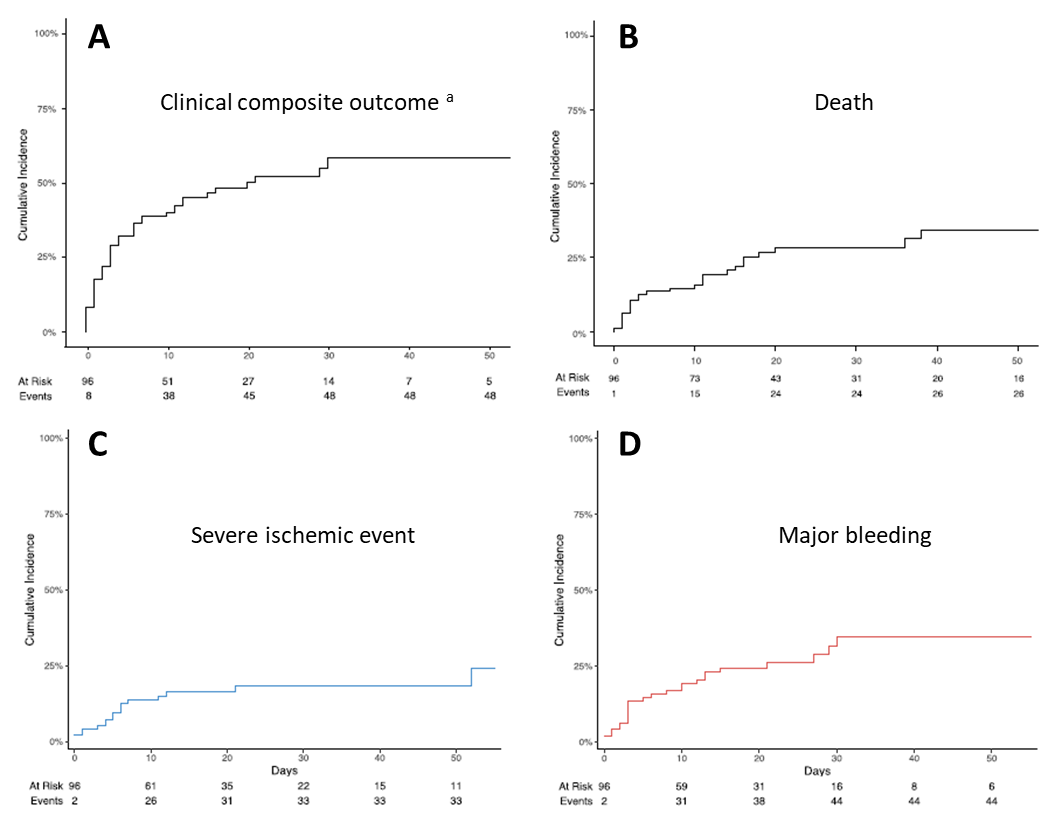


Abbreviations: AMI, acute myocardial infarction

^a^ composite of severe ischemic event (acute myocardial infarction recurrence or stroke), major bleeding, or death from any cause

# References

1. Thygesen K, Alpert JS, Jaffe AS, Chaitman BR, Bax JJ, Morrow DA, et al. Fourth universal definition of myocardial infarction (2018). Eur Heart J. 2018;

2. Vranckx P, White HD, Huang Z, Mahaffey KW, Armstrong PW, Van de Werf F, et al. Validation of BARC Bleeding Criteria in Patients With Acute Coronary Syndromes: The TRACER Trial. J Am Coll Cardiol. 2016;67:2135–44.

3. Thygesen K, Alpert JS, Jaffe AS, Chaitman BR, Bax JJ, Morrow DA, et al. Fourth Universal Definition of Myocardial Infarction (2018). Circulation. 2018;138:e618–51.

4. Antman EM, Cohen M, Bernink PJ, McCabe CH, Horacek T, Papuchis G, et al. The TIMI risk score for unstable angina/non-ST elevation MI: A method for prognostication and therapeutic decision making. JAMA. 2000;284:835–42.

5. Singer M, Deutschman CS, Seymour CW, Shankar-Hari M, Annane D, Bauer M, et al. The Third International Consensus Definitions for Sepsis and Septic Shock (Sepsis-3). JAMA. 2016;315:801–10.

6. Vranckx P, White HD, Huang Z, Mahaffey KW, Armstrong PW, Van de Werf F, et al. Validation of BARC Bleeding Criteria in Patients With Acute Coronary Syndromes: The TRACER Trial. J Am Coll Cardiol. 2016;67:2135–44.
